# Editorial: The role of vitamin D as an immunomodulator

**DOI:** 10.3389/fimmu.2023.1186635

**Published:** 2023-03-28

**Authors:** Mourad Aribi, Franck J. D. Mennechet, Chafia Touil-Boukoffa

**Affiliations:** ^1^ Laboratory of Applied Molecular Biology and Immunology, W0414100. University of Tlemcen, Tlemcen, Algeria; ^2^ Pathogenesis and Control of Chronic and Emerging Infections, The Institut National de la Santé et de la Recherche Médicale (INSERM) U1058, University of Montpellier, Etablissement Français du Sang, Antilles University, Montpellier, France; ^3^ Cytokines and Nitric Oxide (NO) Synthases Team, Laboratory of Cellular and Molecular Biology (LBCM), Faculty of Biological Sciences, University of Sciences and Technology Houari Boumediene (USTHB), Algiers, Algeria

**Keywords:** vitamin D, immunomodulation, transcriptome, gut microbiota, platelet-mediated inflammation, exercise, deep vein thrombosis

## The metabolism and immunomodulatory role of vitamin D: An abbreviated overview

**Figure 1:**
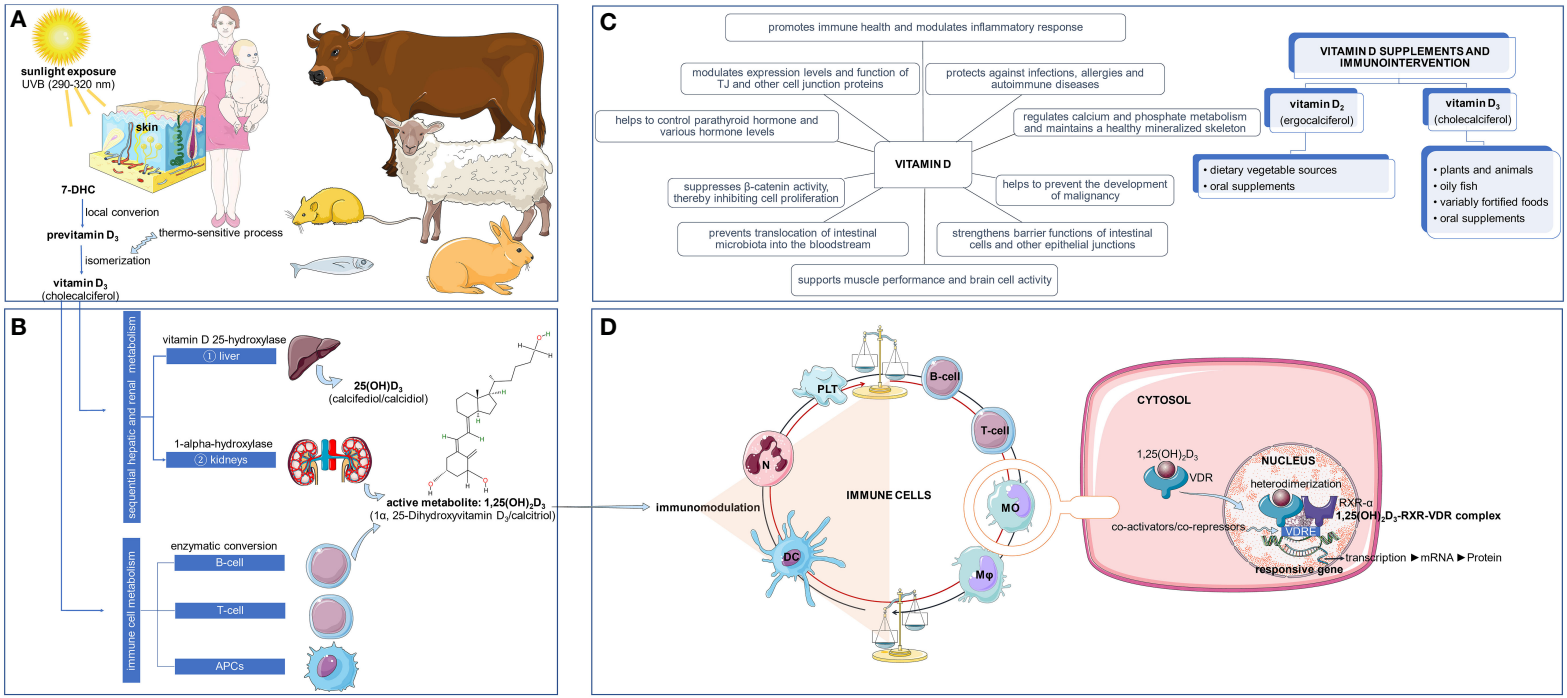
The complexity of vitamin D3: synthesis, metabolism, mechanism of action, and immunointervention. The bioactive form of vitamin D_3_ plays an important role in the modulation of immune system. The skin of humans and some animals synthesize vitamin D_3_ when exposed to ultraviolet B (UVB) radiation (290–320 nm) by acting on 7-dehydrocholesterol (7DHC) **(A)**. After undergoing enzymatic processes in the liver and kidneys, vitamin D_3_ is then metabolized into its active form, 1,25(OH)_2_D_3_ (1α, 25-Dihydroxyvitamin D_3_/calcitriol) by vitamin D 25-hydroxylase and 1-alpha-hydroxylase, respectively. Some immune cells, including B-cells, T-cells and antigen presenting cells (APCs), are also able to transform vitamin D_3_ into its active metabolite through a process of enzymatic conversion. **(B)**. Not only does vitamin D_3_ perform many vital roles, it has been shown to possess powerful effects that can control immune function, allowing it to be utilized in immunologic interventions to keep the immune system functioning optimally and to correct any existing vitamin D deficiencies (C). The immune effects of vitamin D_3_ are ensured by its active metabolite, i.e., 1,25(OH)_2_D_3_. Acting upon specific immune cells, 1,25(OH)_2_D_3_ binds to the vitamin D receptor (VDR) in the cytoplasm and then heterodimerizes with the retinoid X receptor-a (RXR-a) in the nucleus, forming the 1,25(OH)_2_D_3_-RXR-VDR complex which attaches to vitamin D response elements (VDRE) on the DNA **(D)**. DC, dendritic cell; MO, monocyte; Mφ, macrophage; N, neutrophil; PLT, platelet; TJ, tight junction. Some illustrations were sourced from the Servier Medical Art platform.

Considered as a vitamin and a pre-hormone, vitamin D, in its biologically active form (1,25-dihydroxyvitamin D_3_ [1,25(OH)_2_D_3_]) is able to immunomodulate the functional activities of both innate and adaptive immune cells (1), including B-cells (2), T-cells (3), monocytes (4), macrophages (5), dendritic cells (6), neutrophils (7), and platelets (8), as these cells express the vitamin D receptor (VDR), a nuclear receptor and ligand-activated transcription factor/a ligand-dependent transcription regulator molecule belonging to the superfamily of nuclear receptors (9). Mechanistically, the action of the 1,25(OH)_2_D_3_ on the target cells is performed by its binding to the VDR in the cytoplasm, followed by its heterodimerization with the retinoid X receptor-α (RXR-α) in the nucleus, resulting in the formation of the 1,25(OH)_2_D_3_-RXR-VDR complex that binds to vitamin D response elements (VDRE) located on DNA (10) (**Figure 1**).

Humans and some animals have the ability to synthesize vitamin D in the form of previtamin D_3_ in the skin as a result of localized conversion of 7-dehydrocholesterol (7-DHC) when exposed to solar ultraviolet B rays (UVB, 290-320 nm) (11–13). Previtamin D_3_ isomerizes to vitamin D_3_ (cholecalciferol) in the skin, in a thermo-sensitive but noncatalytic process (14), and both previtamin D_3_ and vitamin D_3_ are converted into a variety of photoproducts thanks to UVB radiation (15). Vitamin D_3_ is considered to be best metabolized by the body. Its metabolism takes place consecutively in the liver, where it undergoes a first hydroxylation at the carbon 25-position into calcifediol/calcidiol ([25(OH)D_3_]) by the enzyme microsomal vitamin D 25-hydroxylase, also known as a cytochrome p450 2R1, which is encoded in humans by the CYP2R1 gene (16), and then, according to the cell needs and in response to cellular signals, it is hydrolyzed mainly in the kidneys at the 1α-position into 1,25(OH)_2_D_3_, *i.e.*, calcitriol (17) by the enzyme 25-hydroxyvitamin D 1-alpha-hydroxylase (VD 1A hydroxylase), referred to as cytochrome p450 27B1 (CYP27B1)/1-alpha-hydroxylase, which is encoded by the CYP27B1 gene in humans (18). As VDRs, the most vitamin D metabolizing enzymes are present in various immune cells, including T-cells, B-cells and antigen-presenting cells (APCs) (19).

## Vitamin D deficiency and potential immunomodulatory interventions

Although the bioactive form of vitamin D is calcitriol, its status is assessed by determining the concentration of the so-called “circulating/blood” vitamin D form which corresponds to the result of the first vitamin D conversion, *i.e.*, calcifediol. In addition, it should be accepted that there are no single criteria and methods defining normal values for vitamin D. Nevertheless, it has been suggested that the normal levels of circulating blood vitamin D in human measured in late winter or early spring is greater than or equal to 75 nmol/L (30 ng/mL) as a cutoff value (20). While, greater than 20 ng/mL as optimal level, 11-20 ng/mL as insufficient, and vitamin D deficiency has been defined by a level less than or equal to 10 ng/mL (to convert to nmol/L, multiply by 2.496) (21).

Vitamin D deficiency may expose to increased risk of developing various immune-related diseases, including systemic and organ-specific autoimmune diseases, tuberculosis, sepsis, respiratory infection, and COVID-19, etc. (22, 23). This appears to be generalized in many geographical localities, and can be considered as a global public health problem in all age groups (24). This involves (i) insufficient sun exposure levels during the winter period (25), (ii) air pollution and low ground level of UVB rays as a result of overcast clouds (iii) a diet poor in vitamin D_2_ (ergocalciferol) and vitamin D_3_, (iv) aging, as it affects the formation of 1,25 (OH)_2_D_3_ (26), and (v) pigmented skin, as melanin absorbs the UVB that initiates vitamin D synthesis (27), etc.

Two bioequivalent forms of vitamin D can be recommended as vitamin D supplements, in order to correct such a deficiency (28), and include vitamin D_2_ (obtained from dietary vegetable sources and oral supplements) and vitamin D_3_ (present in plants and animals and obtained from oily fish and variably fortified foods and oral supplements) (29). In this context, they may also be the subject of nutritional immunomodulation intervention, since vitamin D has been shown to play a key role in stimulating immune responses, and protecting against infections, including respiratory tract infection (30), allergies (31), auto-immune diseases (32), and the development of malignant tumors (33). Vitamin D can also strengthen the barrier function of intestinal cells and different epithelial junctions, thus preventing the translocation of the intestinal microbiota to the bloodstream. Finally, vitamin D can suppresses the activity of β-catenin and then inhibits cell proliferation, modulates the expression level and functions of tight junction (TJ) proteins, such as claudin2 and 12, and other cell junction proteins, including E-cadherin, Occludin and ZO-1 (34).

## The role of vitamin D as an immunomodulator: A review of the key findings from the research topic

This Editorial looks at the key findings related to *The Role of Vitamin D as an Immunomodulator* that have been announced in our Research Topic. It covers a compilation of six papers that were given precedence for publication within the topic by diverse research teams.

The Carlberg team has been able to gain knowledge on how vitamin D_3_ supplementation affects immunity by examining the transcriptome of peripheral blood mononuclear cells (PBMCs) when stimulated with either lipopolysaccharide (LPS) and β-glucan (BG), both in the presence or absence of 1,25(OH)_2_D_3_. Their findings point out that while 1,25(OH)_2_D_3_ does affect immune responses, its effect is largely dependent on when it is administered regarding a real-life situation where vitamin D_3_ has been supplemented after, before or during an infection. This evidence should be beneficial to clinicians as this indicates that timing plays a huge role in considering vitamin D_3_ supplementation. Additionally, the findings of Carlberg and colleagues provide further proof for the role of vitamin D_3_ in supporting a healthy immune system and could give insight into when and how it can be used to treat or prevent certain illnesses through altering functional consequences of immune challenges. These results should have substantial implications for medical practice, thus warranting more studies to support them.

The human blood transcriptome is an important factor in the understanding of immune system health and function. Despite this, there has been a lack of research into how vitamin D_2_ and D_3_ supplementation affects it. Durrant et al. have addressed this gap by conducting an extensive study on the effects of physiological doses of these vitamins on the human blood transcriptome using microarrays. They sought to gain greater insight into this question by extending an earlier study exploring changes in the blood transcriptome of healthy European and South Asian women who were given daily doses of either vitamin D_2_ or D_3_ over 12 weeks during wintertime in the UK. Their research provides valuable insight into how the two forms of vitamin D can affect gene expression within individuals over time, which may have implications for the treatment of a variety of conditions, including influence of ethnic background. Their study provides compelling evidence for the differential effects of vitamin D_2_ and D_3_ on gene expression, including gene expression associated with type I and type II interferon activity, and highlights potential differences between ethnicities. The results suggest that further research is needed to determine the physiological roles of each form of vitamin D, as well as their effects on gene expression among different populations. This ground-breaking research offers important insights into the molecular mechanisms underpinning the physiological roles of each form of vitamin D, highlighting potential differences between them. This study is particularly timely given increasing rates of both deficiency and supplementation with either or both forms of vitamin D across many populations worldwide. The findings have important implications for understanding how these two forms may interact differently at a cellular level to modulate immune responses, potentially leading to different outcomes when considering disease susceptibility, severity or treatment efficacy. In conclusion, this work serves as an essential foundation upon which to build further research into the cellular and molecular mechanisms by which vitamin D_2_ and D_3_ differentially influence gene expression, ultimately impacting on immune responses. It is clear that a greater understanding of this area is needed to inform clinical management strategies for individuals at risk of deficiency or requiring supplementation. An improved comprehension of how these two forms may interact differently could lead to more effective treatments and interventions in many diseases associated with dysregulation of the immune system.

The intricate relationship between exercise and immunity is one that has been explored by scientists for many years. It is now becoming increasingly clear that physical activity, intensity of effort, as well as dietary factors like vitamin D can all play a role in modulating our immune system. Thanks to a narrative review, Crescioli highlights how vitamin D shapes human immunity through its effects on both the immune system and skeletal muscle in athletes, who are particularly exposed to oxidative stress and inflammation due to intense training. She highlights the pros and cons associated with its immunomodulatory properties and outlines future research directions for further investigation into this important topic. Therefore, several aspects have been reported, including more specifically effort intensity effect on immunity, vitamin D-induced regulation of skeletal muscle immunity, and vitamin D’s effect on muscular immunity, etc. Overall, physical activity should be monitored closely when training at high intensities; additionally, athletes should strive for adequate levels of vitamin D intake as well as regular sun exposure in order to maintain a healthy immune system. Further research is needed to better understand the relationship between exercise, vitamin D, and immunity; however, some important findings should be considered for developing new and more adequate training regimens for athletes.

It has been established that deep vein thrombosis (DVT) is a major health issue around the world. Risk factors for DVT include age, extended periods of immobility or being confined to a wheelchair due to medical issues such as cancer and heart disease, hormone replacement therapy, post-surgery complications, obesity, smoking and taking medications containing estrogen. People who fly often may also be at an increased risk since they spend long stretches without moving. Treatment usually involves anticoagulant drugs which prevent clotting but can have serious side effects. There is now evidence that vitamin D plays an important role in preventing DVT; people with lower levels of vitamin D are more likely to experience it. This inspired Tao et al. to investigate whether the gender gap in DVT and vitamin D levels might explain why female stroke patients have a greater chance of developing DVT due to their lower vitamin D levels. Their research has found that women are more likely than men to develop DVT, and this could be related to differences in their vitamin D levels. The implications of this study suggest further investigation into whether providing extra vitamin D through supplementation can reduce the risk of DVT, especially for female stroke patients.

The relationship between vitamin D, the immune system, and the gut microbiota has not been extensively researched yet; therefore, there is not much knowledge on how taking vitamin D supplements impacts intestinal traits in individuals prone to autoimmune diseases. Martens et al. conducted a study to explore the long-term effects of varying levels of dietary vitamin D supplementation (400 and 800 IU/day) on type 1 diabetes (T1D) development in nonobese diabetic (NOD) mice, as well as its link with 25(OH)D_3_ concentration levels and their impact on peripheral/gut immunity, exploring specifically FoxP3^+^ Treg cells, CD39^+^CD73^+^ T-cells, IL-10-secreting T-cells, and Tr1 cells, intestinal barrier function, and gut microbiota composition. Results showed that the 800 IU/day dose was associated with delayed disease onset; significantly decreased incidence of T1D; increased rate of Treg cells in various lymphoid organs; and a modified intestinal microbiota signature, with an increase in *Ruminiclostridium_9* and decrease of *Marvinbryantia* at the genus level. Despite this research, more studies are needed to gain further knowledge on how taking vitamin D supplements impacts intestinal traits in people prone to T1D and other autoimmune diseases.

Although vitamin D supplementation has been shown to have potential anti-inflammatory effects by reducing platelet-mediated inflammation, its effects on platelet-mediated inflammation in patients with type 2 diabetes (T2D) have yet to be sufficiently studied. Thus, Johny et al. conducted a single-center, randomized, double-blind, placebo-controlled study to evaluate the potential impact of vitamin D supplementation on platelet activation and systemic inflammation in patients with T2D who were deficient in vitamin D. The trial was supported by an extensive analysis of clinical and biochemical variables, vitamin D binding protein (VDBP), immunome profiling, platelet–immune cell aggregate, oxidative and nitrosative stress biomarkers as well as pro- and anti-inflammatory cytokines and chemokines. The results suggest that supplementing vitamin D may be beneficial for T2D patients by alleviating oxidative stress and reducing platelet-mediated inflammation - thereby potentially slowing down the progression of the disease and decreasing cardiovascular risk.

## Conclusions

In conclusion, the current Research Topic yielded important knowledge regarding the complexity of the connection between vitamin D, human health and immunity. It has highlighted that vitamin D can have a wide range of impacts, including influencing gene expression, modulating immune response, inflammation, oxidative stress, and the gut microbiota signature. These findings suggest that vitamin D supplementation may be beneficial in the treatment or prevention of a variety of illnesses and conditions. Nevertheless, further research into the molecular and cellular mechanisms of vitamin D is warranted to substantiate these claims and to further explore the intricate relationship between vitamin D and human immunity in health and disease.

## Author contributions

MA made a significant impact on the progression and presentation of the Research Topic, including actively aiding in the writing of the text, preparing the manuscript, designing the Editorials’ accompanying Figure, and providing analyses and conclusions featured in the articles included in this Research Topic. FM was instrumental in the formation and organization of the Research Topic, aiding in the writing of the text and the preparation of the manuscript. CT-B conducted thorough research on the topic and supplied key concepts for the manuscript, while also supplying helpful data to the Research Topic proposal. All the authors were actively involved in the editing of the articles submitted for publication.
